# Development and validation of the provider assessed quality of consultations with language interpretation scale (PQC-LI)

**DOI:** 10.1186/s13104-023-06675-7

**Published:** 2024-01-04

**Authors:** Frank Müller, Julie Ngo, Judith E. Arnetz, Harland T. Holman

**Affiliations:** 1 Department of Family Medicine, Michigan State University, 15 Michigan St NE, Grand Rapids, MI 49503 USA; 2 Spectrum Health Family Medicine Residency Center, 25 Michigan St NE Suite 5100, Grand Rapids, MI 49503 USA; 3 Department of General Practice, University Medical Center Göttingen, Humboldtallee 38, 37073 Göttingen, Germany

**Keywords:** Language barrier, Migrant, Limited English language proficiency, Questionnaire, Patient-centeredness, Interpreters, Language interpretation, Shared-decision making, Consultation, General practice, Outpatient care

## Abstract

**Objective:**

With the growing immigrant communities in the western world, there is an urgent need to address language barriers to care, and health disparities as a whole. Studies on limited English proficiency patients (LEP) have focused on patient perspectives of office visits, however little is known about health care provider perspectives of medical visits using interpretive services. We aimed to develop a pragmatic brief questionnaire for assessing providers’ views of the quality of communication in outpatient visits with patients with LEP. The questionnaire was validated in a cross-sectional study (n = 99) using principal component analyses (PCA) with oblimin rotation. Internal consistency was analyzed using Cronbach’s alpha.

**Results:**

Based on theory and literature, a seven-item scale was developed that captures two relevant concepts: (1) Provider - patient interaction during the consultation and (2) perceived quality of translation. The questionnaire was used to assess 99 LEP consultations and demonstrated good feasibility in a clinical setting. PCA revealed the two theory-based components with good factor loadings and internal consistency of α = 0.77. These preliminary results indicate that the questionnaire provides medical professionals with a validated tool to evaluate LEP patient encounters. Further confirmatory validation of the Provider-assessed Quality of Consultations with Language Interpretation (PQC-LI) in larger samples is warranted.

**Supplementary Information:**

The online version contains supplementary material available at 10.1186/s13104-023-06675-7.

## Introduction

Increasingly, family physicians in the US and other western countries are providing care to patients with limited proficiency in the locally spoken language [1]. The United States Census Bureau estimates that approximately 20% of the U.S. population speaks a language other than English at home [2]. Patients with limited English language proficiency (LEP) experience barriers in accessing health services [3], and have poorer health outcomes in many different areas, even if controlled for sociodemographic disparities [4–6]. Language interpretation is the key to overcoming language barriers during visits [7]. Furthermore, there are US federal laws that prohibit discrimination in health care programs and make it mandatory for health care providers to have language interpretation services available [8]. Various studies have measured LEP patients’ satisfaction with language interpretation [7, 9–11], for example, between in-person and video interpreters [12]. Other studies used instruments that measure patient-centeredness and shared decision-making in outpatient encounters [13–15], some of which focused on consultations with LEP patients and interpreters [16, 17]. However, there is no validated instrument that reflects the provider’s perspective on consultation with language interpretation. Considering the fact that both patient and provider perspectives are needed to best understand and evaluate care consultations [18], our aim was to develop such a tool. As part of a comprehensive quality improvement project to assess the challenges and perceptions of patients, providers, and interpreters in encounters with LEP patients, we have developed and validated a short and pragmatic instrument.

## Main text

### Methods

#### Study design and participants

This is a cross-sectional study conducted in a large urban family medicine clinic with currently 16 providers in Grand Rapids, Michigan. Between June 7th 2022 and August 15th 2022 the appointment calendar was screened for LEP encounters. Before each appointment, questionnaires were distributed to the providers. As providers often worked either one or two half-day shifts, questionnaires were typically filled out either right after seeing the patient or before break / leaving, reducing the timespan between patient contact to survey completion to < 4 h and likewise reducing recall bias.

#### Questionnaire development

The first step in developing the questionnaire was to review the literature on existing instruments for assessing patient and provider satisfaction in consultations with and without interpreters. We identified two relevant survey components: (1) Provider - patient interaction during the consultation [19] and (2) perceived quality of translation [20]. Based on these theoretical constructs and on items from other survey instruments, seven items were developed in a discursive process among the author team that includes researchers and three family physicians of various career stages who provide primary care to LEP patients on a regular basis. Some items were inspired by other patient-administered questionnaires to rate their providers, e.g. “Did the doctor allow you enough time to explain the reasons for your visit?” [9] but changed to a provider perceived perspective. The first questionnaire draft was initially piloted in 8 consultations. Researchers wanted to see if the questionnaire would suit different types of visits, such as well-child examinations, physicals, or visits with acute complaints. Additionally, we focused on comprehensibility and completion time. The questionnaire was finalized with a further round of minor adjustments and is provided as an appendix to this article.

#### Measurements

The questionnaire consists of 7 items that can be answered on a 5-point Likert scale ranging from “yes” (= 0) to “no” (= 4). With the exception of the first item (“I gave the patient enough room to explain their concerns”), all items describe difficulties that the provider may have encountered (e.g., “I felt like I was rushing”). To calculate a total score, the score of the first item was inversed and summed up with all other items. Scores range from 0 to 28, with higher scores indicating a better quality of consultation. We hypothesized that items 1–5 would comprise the first subscale (patient - provider interaction) with sum-scores ranging from 0 to 20, and items 6–7 would comprise the second subscale (perceived quality of translation) with sum-scores ranging from 0 to 8.

In addition to collecting the questionnaires completed by the providers, we extracted patient sociodemographic (age, gender, preferred language) and consultation-related information (provider, type of appointment, duration of encounter) from patient charts.

### Statistical analyses

Participant characteristics and questionnaire sum-scores were assessed using absolute and relative numbers, mean, standard deviation and median. Distribution of sum-scores were analyzed using kurtosis and skewness. Bivariate statistics were applied to compare patient demographic characteristics in encounters with and without available provider questionnaires. Questionnaire sum-scores were also examined by patient demographics using Chi-Square test, Mann-Whitney-*U* test and Spearman’s correlation, where appropriate. Internal consistency was assessed for the overall score and subscales using Cronbach’s alpha (α). Kaiser-Meyer-Olkin-Criteria (KMO) and Bartlett’s test of sphericity were used to assess item set appropriateness for the principal component analysis (PCA) [21, 22]. A PCA with oblimin rotation (delta = 0) and scree plots was used to extract factors and factor loadings. Items were tested whether they had a suitable explanatory power using item-total correlation thresholds of < 0.3 and factor loadings < 0.4. Further, items were checked for inter-item collinearity using a threshold for correlation coefficient > 0.8. Other research has suggested an á priori item to response ratio of 1:10 for most PCAs [23] resulting in a sample size of least n = 70 responses to sufficiently power our study. All analyses were performed using SPSS Version 27 (IBM, Armonk, NY).

### Ethic

This study was originally conceived as quality improvement by the Spectrum Health institutional review board (Decision# 2021 − 169) but eventually converted into a research project which underwent full review and was approved by the Spectrum Health institutional review board (Decision# 2022 − 375). Informed consent was obtained from all providers participating in the study. No compensation was granted to participants.

## Results

Out of 155 encounters with LEP patients during the two-month study period, 99 provider-completed questionnaires (64%) were returned and analyzed. The questionnaires were completed by a total of 16 providers comprised of 4 attending physicians, 11 residents, and 1 nurse practitioner. Eight providers identified as male, 7 as female, and 1 as non-binary. The item to response ratio was 1:14.1. Patient and provider demographics and characteristics of encounters are shown in Table 1. A larger proportion of consultations with returned questionnaires concerned treatment of patients with acute conditions (76.8% vs. 57.1%, p < 0.05) and patients who spoke Burmese language (21.2% vs. 16.1%, p < 0.01). Otherwise, no other differences between groups were noted.

**Table 1:** Characteristics of LEP encounters (n = 155) with and without provider questionnaire responses

		Questionnaire available		
		yes (n = 99)	no (n = 56)	
		n (%)	n (%)	p
Patient age	Years, Mean (SD)	30 (21)	32 (22)	0.556
Patient gender	Female	58 (58.6)	31 (55.4)	0.696
	Male	41 (41.4)	25 (44.6)	
Type of visit	Well Child Visit	13 (13.1)	16 (28.6)	0.029
	Acute / Established	76 (76.8)	32 (57.1)	
	Physical	10 (10.1)	8 (14.3)	
Patients preferred language	Spanish	12 (12.1)	21 (37.5)	0.002
	Burmese / Karen	21 (21.2)	9 (16.1)	
	Kinyarwanda	31 (31.3)	16 (28.6)	
	Other	35 (35.4)	10 (17.9)	
Type of interpreter	Lay interpreter	7 (7.6)	n/a	n/a
	In person	27 (29.3)	n/a	
	Phone	40 (43.5)	n/a	
	Video	18 (19.6)	n/a	
Provider’s gender	Female	34 (34.3)	17 (30.4)	0.464
	Male	53 (53.5)	35 (62.5)	
	Non-Binary	12 (12.1)	4 (7.1)	
Type of provider	Attending physician	28 (28.3)	13 (23.3)	0.748
	Resident physician	56 (56.6)	35 (62.5)	
	Nurse practitioner	15 (15.2)	8 (14.3)	
Time spent with provider	Minutes, Mean (SD)	47 (36)	42 (20)	0.820

LEP = Limited English Proficiency

The 99 encounters had PQC-LI sum scores ranging from 2 to 28 with a mean of 23.4 (SD 4.9) and median of 24 points. Skewness of the sum score was found to be -1.3 and kurtosis was 2.7 indicating a left-skewed and tailed distribution compared to normal distribution. Mean scores for each item and the two questionnaires subscales are displayed in Table 2. The PQC-LI had a Cronbach’s α = 0.77. The component (1) provider - patient interaction had α = 0.82 and (2) satisfaction and perceived quality of translation had α = 0.76 indicating good internal consistency. Excluding items did not improve internal consistency.

**Table 2:** Mean scores of items, subscales and overall PQC-LI scale

Item	Mean	SD
Subscale (1): Provider - Patient interaction (Cronbach’s alpha 0.82)	16.5	4.0
1. I gave the patient enough room to explain their concerns	3.8	0.5
2. I felt like I was rushing	3.3	1.1
3. It took me some time to get to the point with the patient	2.8	1.4
4. I may have missed something	3.4	0.9
5. I felt that the patient had difficulty addressing his / her concerns	3.3	1.1
Subscale (2): Satisfaction and perceived quality of translation (Cronbach’s alpha 0.76)	6.8	2.1
6. I felt that the interpreter was adding or missing something	3.5	1.1
7. I felt frustrated with the interpreter and/or the interpreter service	3.4	1.3
Sum score PQC-LI (Cronbach’s alpha 0.77)	23.4	4.9

PQC-LI = Provider-assessed Quality of Consultations with Language Interpretation

All 7 items showed item-total correlation > 0.3 with correlation coefficients ranging between 0.33 and 0.66 and item-item correlations < 0.8 with correlation coefficients ranging between 0.06 and 0.72. Therefore, all items met statistical eligibility criteria.

The questionnaire showed a reasonable sampling adequacy with a Kaiser-Meyer-Olkin statistics value of 0.74. Bartlett’s test of sphericity was significant (χ²(21) = 275.8, p < 0.001). The PCA revealed the two theoretical-based components of the questionnaire (1) provider - patient interaction (5 items) and (2) satisfaction and perceived quality of translation (2 items) using eigenvalue > 1 as cut off. Factor loadings using oblimin rotation are shown in Table 3.

**Table 3:** Factor loadings based on the rotated component matrix

	Component^*^	
	1	2
I gave the patient enough room to explain their concerns	0.504	
I felt like I was rushing	0.915	
It took me some time to get to the point with the patient	0.752	
I may have missed something	0.854	
I felt that the patient had difficulty addressing his / her concerns	0.803	
I felt that the interpreter was adding or missing something		0.924
I felt frustrated with the interpreter and/or the interpreter service		0.885

^*^Factor loadings < 0.4 are omitted

Older patient age was correlated with lower scores on the questionnaire subscale for provider-patient interaction (r=-0,24, p = 0.016) but not with perceived quality of translation. Patient gender was not associated with the overall PQC-LI nor the two subscales. Lower PQC-LI scores were inversely correlated with a longer time span that providers spent with patients (r=-0.27, p = 0.007).

## Discussion

We have developed and validated a brief questionnaire that assesses provider perspectives on consultations with LEP patients with language mediation by interpreters. It can be used in various clinical settings and can be a pragmatic, easy to administer, and useful tool to monitor provider perceived quality of communication with LEP-patients. The tool and the provided baseline data can be further used to identify structural shortcomings in interpretation quality or to define and characterize particular challenging communication situations with LEP patients in primary care. It can be also used in interventional studies to monitor changes in perceived communication quality. With a Cronbach’s α of 0.77, it shows good internal consistency, has two clearly distinguishable and relevant subscales, and with seven items the PQC-LI is sufficiently concise and feasible to implement research in a clinical setting. The validation of the questionnaire was done in particular on consultations with Kinyarwanda and Burmese speaking patients. Patients of excluded consultations did not significantly differ in age, gender, length of time spent with their provider, provider type and provider’s gender.

To our knowledge, this is the first attempt to create and validate a specific provider-administered instrument to focus communication quality on LEP encounter level. Other research has focused on providers’ overall perception of LEP encounters [24], on patient perspectives with more comprehensive instruments (e.g. Interpersonal Processes of Care Survey with 29 items [25]), or researcher developed unvalidated instruments [26].

The first subscale of the PQC-LI is meant to capture whether providers faced difficulties in obtaining relevant information and providing a patient-centered atmosphere during the encounter. Patient centeredness can be considered a challenge in providing care for LEP patients. LEP patients mention symptoms, feelings, expectations and thoughts less frequently [27] and providers are more likely to ignore patient’s comments than in encounters with non-LEP patients [19]. This may result in worse interpersonal care [9] and a communication style that leads to less shared-decision making [28, 29]. The second subscale of the PQC-LI measures the perceived quality of the translation and satisfaction with the interpreter. As quality of interpretation may vary, it can introduce misunderstanding [7, 20, 30]. Additionally, interpreter’s attitudes and their assumed role during consultation is decisive in enabling a trustworthy patient-provider relationship [31]. The PCA revealed a clear distinction between the two subscales. Although the two item subscale is uncommon [32], it is theory-based and it showed good reliability and explanatory power for the overall scale.

The instrument requires that providers are able to reflect on consultations, which may vary between providers and may introduce a bias. Future research should also address patients’ perspectives, and examine whether their perceptions align with those of the providers.

On average, providers who responded to the questionnaire spent 5 min longer with their patients than those who did not respond; this time difference was not statistically significant. Nevertheless, it was surprising that we found an inverse correlation between overall PQC-LI scores and consultation time. This may reflect a greater frustration with the patient encounter among providers whose consultations took longer. While substantial research has shown that length of time spent with the physician is an important predictor of patient satisfaction [33], less is known about physician satisfaction with consultation length. Despite an existing ceiling effect, the questionnaire can be used to identify consultations in which communication was particularly challenging.

### Limitations

Our PQC-LI comes with limitations. While feasibility in daily practice has shown to be an advantage, it will not cover the entire complexity of communication in LEP encounters. Analysis of criterion validity was not possible, as no gold standard for provider perceptions of LEP encounters yet exists. The PQC-LI is provider self-reported and covers the subjective perspective. It may not reflect the patient’s or interpreter’s perspective. In future research, specific instruments designed for patients/interpreters should be combined with the PQC-LI for a more comprehensive perspective. Typically for a pilot study, our instrument has only been studied in a small cohort within a single organization. Transferability to other settings or practices is pending. The role of PQC-LI scores in clinical outcomes will be the subject of further investigation.

### Electronic supplementary material

Below is the link to the electronic supplementary material.

Supplementary Material 1: Appendix

## Data Availability

Analyzed data tables are available from the authors on reasonable request and within a data sharing agreement.

## Abbreviations

KMO: Kaiser-Meyer-Olkin-Criteria
LEP: Limited English Proficiency
PCA: Principal component analyses
PQC-LI: Provider-assessed Quality of Consultations with Language Interpretation
SD: Standard Deviation
US: United States of America
