# Supplementary material for: Development and validation of the provider assessed quality of consultations with language interpretation scale (PQC-LI)
Source: BMC Res Notes. 2024 Jan 4;17:15. doi: 10.1186/s13104-023-06675-7 (PMC10768141; doi:10.1186/s13104-023-06675-7)
Supplement: Supplementary file 1 — Supplementary Material 1: Appendix [file 13104_2023_6675_MOESM1_ESM.docx]

# Appendix

A1: Provider assessed quality of consultations with language interpretation Scale (PQC-LI)

**During the visit:**

|  |  | yes |  | partially |  | no |
| --- | --- | --- | --- | --- | --- | --- |
| 1 | I gave the patient enough room to explain their concerns | 🞏 | 🞏 | 🞏 | 🞏 | 🞏 |
| 2 | I felt like I was rushing | 🞏 | 🞏 | 🞏 | 🞏 | 🞏 |
| 3 | It took me some time to get to the point with the patient | 🞏 | 🞏 | 🞏 | 🞏 | 🞏 |
| 4 | I may have missed something | 🞏 | 🞏 | 🞏 | 🞏 | 🞏 |
| 5 | I felt that the patient had difficulty addressing his / her concerns | 🞏 | 🞏 | 🞏 | 🞏 | 🞏 |
| 6 | I felt that the interpreter was adding or missing something | 🞏 | 🞏 | 🞏 | 🞏 | 🞏 |
| 7 | I felt frustrated with the interpreter and/or the interpreter service | 🞏 | 🞏 | 🞏 | 🞏 | 🞏 |
